# Matriz de riesgo para estimar brotes importados de sarampión o rubéola aplicada a Chile

**DOI:** 10.26633/RPSP.2017.47

**Published:** 2017-11-17

**Authors:** Doris Gallegos, Natalia Vergara, Luz Gatica, Carlos Castillo, Andrea Basaldúa, Rodrigo Guerrero, Pamela Bravo-Alcántara, Roberto del Aguila, Sergio Loayza

**Affiliations:** 1 Departamento de Epidemiología, División de Planificación Sanitaria Subsecretaría de Salud Pública, Ministerio de Salud Santiago Chile Departamento de Epidemiología, División de Planificación Sanitaria, Subsecretaría de Salud Pública, Ministerio de Salud, Santiago, Chile.; 2 Geógrafos Asesores técnicos del estudio Comuna de Santiago Chile Geógrafos, Asesores técnicos del estudio, Comuna de Santiago, Chile.; 3 Organización Panamericana de la Salud Washington, DC Estados Unidos de América Organización Panamericana de la Salud, Washington, DC, Estados Unidos de América.

**Keywords:** Public health, geography, medical, geographic information systems, risk management, Chile, Salud pública, geografía médica, sistemas de información geográfica, gestión de riesgos, Chile, Saúde pública, geografia médica, sistemas de informação geográfica, gestão de riscos, Chile

## Abstract

**Objetivo.:**

Desarrollar una matriz de riesgo para evaluar el riesgo continuo de brotes de sarampión y rubéola asociados con la importación de casos en Chile.

**Métodos.:**

El desarrollo de herramientas de evaluación de riesgos se realizó en las siguientes etapas: preparación y aprobación de variables biológicas, programáticas y demográficas, ponderación por un panel de expertos de las variables seleccionadas, cálculo del índice de riesgo, espacialización, y transferencia de conocimiento.

**Resultados.:**

De las 346 comunas de Chile analizadas, 34% se encontraba en el intervalo de riesgo alto de desarrollar un brote de sarampión y rubéola si se producía la introducción del virus, 59%, en el intervalo de riesgo medio, y 3%, en el intervalo de riesgo bajo. El porcentaje restante correspondió a comunas carentes de datos en al menos una de las trece variables requeridas para el cálculo del índice de riesgo.

**Conclusión.:**

La utilización de esta herramienta permitirá a los equipos subnacionales emplear sus propios datos para evaluar el riesgo de brotes en sus áreas y realizar acciones correctivas para responder rápidamente a cualquier importación de virus en la fase posterior a la eliminación.

## Abstract (en)

***Objective*.:**

Develop a risk matrix to evaluate the ongoing risk of measles and rubella outbreaks associated with imported cases in Chile.

***Methods*.:**

The risk assessment tools were developed in the following stages: preparation and approval of biological, programmatic, and demographic variables; weighting of the selected variables by a panel of experts; calculation of the risk index; specialization; and knowledge transfer.

***Results*.:**

Of the 346 Chilean communes analyzed, 34% were in the high-risk interval for experiencing a measles and rubella outbreak with the introduction of the virus, 59%, in the average-risk interval, and 3%, in the low-risk interval. The remaining percentage corresponded to communes lacking data in at least one of the 13 variables required for calculating the risk index.

***Conclusion*.:**

Use of this tool will enable subnational teams to use their own data to evaluate the risk of outbreaks in their area and take corrective action for a rapid response to any importation of these viruses in the post-elimination phase.

## RESUMO

Desenvolver uma matriz de risco para avaliar o risco contínuo de surtos de sarampo e rubéola associados com a importação de casos no Chile.

As ferramentas de avaliação de riscos foram desenvolvidas nas seguintes etapas: preparação e aprovação das variáveis biológicas, programáticas e demográficas, consideração das variáveis selecionadas por painel de especialistas, cálculo do índice de risco, especialização e transferência de conhecimento.

Das 346 comunidades analisadas, 34% estavam dentro da faixa de alto risco de ter um surto de sarampo e rubéola com a introdução do vírus, 59% na faixa de risco intermediário e 3% na faixa de baixo risco. O percentual restante correspondeu a comunidades com dados insuficientes em pelo menos uma das 13 variáveis necessárias ao cálculo do índice de risco.

***Conclusão*.:**

A utilização desta ferramenta permitirá às equipes subnacionais lançar mão de dados próprios para avaliar o risco de surtos e realizar medidas corretivas para responder rapidamente a qualquer importação de vírus na fase posterior à eliminação.

En los últimos años han reaparecido enfermedades transmisibles que se habían controlado a nivel mundial. Como resultado de la globalización y del consecuente aumento de las migraciones, la incidencia de determinadas enfermedades ha aumentado y se han declarado brotes, lo que constituye un problema de salud pública a pesar de que existen medidas efectivas para su prevención y control. En este contexto destacan enfermedades graves y altamente contagiosas como el sarampión y la rubéola, tanto en países en desarrollo como en países desarrollados (1).

Con las altas coberturas de vacunación alcanzadas por Chile y en la Región de las Américas con el programa regular y campañas se lograron eliminar ambas enfermedades (2). Así, la Organización Panamericana de la Salud (OPS) certificó en 2015 la eliminación de la rubéola (3) y del síndrome de rubeola congénita, y en 2016, del sarampión (4).

El Ministerio de Salud (MINSAL) de Chile indica que, mientras no se concrete la erradicación mundial del sarampión y la rubéola, se mantendrá la circulación de estos virus y, por lo tanto, en la Región continuarán importándose casos con el consecuente riesgo de brotes en los países (5), como ha ocurrido, entre otros países, en los Estados Unidos de América, Canadá y Brasil (6). En 2015, en Chile se confirmó un brote de sarampión con 9 casos en la capital del país asociado con la importación (genotipo vírico H1) (7). En ese mismo año, en Perú se produjo otro brote importado desde Alemania, también con un limitado número de casos, una situación que se repitió en otros países (6).

Los países tienen la obligación de mantener los logros alcanzados, incluido el desafío de certificar la eliminación de estas enfermedades en la Región y estar preparados frente a potenciales riesgos de reintroducción de estos virus (3–5). Con las estrategias de Chile para mantener la eliminación del sarampión y la rubéola y establecer la vigilancia activa sindrómica e integrada de ambas enfermedades se desarrolló una herramienta epidemiológica de apoyo para la toma de decisiones, definida como matriz de riesgo, cuyo formato de origen se ajustó a los requisitos del país. Los resultados obtenidos se presentaron en 2014 y 2015 (8).

Es así como desde la geografía de la salud y sus subespecialidades se reconoce que la identificación de áreas de riesgo a escala comunal permite concentrar los recursos y los esfuerzos en la prevención de enfermedades, mejorar los procesos y asignar recursos a la salud pública para la toma de decisiones (9–10).

El presente estudio se centra en describir el método de adaptación de la matriz de riesgo con variables dicotómicas a una estandarizada, perfeccionando la captura de información y la construcción de la base de datos disponible en Chile a escala comunal. La comuna se define como la unidad territorial básica de la estructuración del Estado de Chile en que se divide la provincia y cuya autoridad jurisdiccional es la municipalidad.

En un escenario de posteliminación de estas enfermedades en la Región, el objetivo de este artículo es describir el método de elaboración de una matriz de riesgo que permite estimar la probabilidad de brotes asociados con la importación de casos de sarampión o rubéola en Chile para que las zonas de riesgo medio y alto prioricen sus intervenciones para mejorar sus índices y los resultados en salud.

## MATERIALES Y MÉTODOS

### Modelo base de la matriz de riesgo

La matriz de riesgo es una herramienta de control y gestión utilizada para identificar el tipo, grado de amenaza y vulnerabilidad inherente a las actividades de un programa, así como los factores exógenos y endógenos relacionados (8). La flexibilidad de esta herramienta es una de sus características más importantes para poder adaptarla de acuerdo con los requerimientos establecidos y las necesidades en cada momento. Por su parte, los sistemas de información geográfica que se asocian con la construcción de la matriz permiten a los usuarios analizar la información espacial, editar datos, construir mapas y presentar los resultados de forma amigable e interactuar con ellos. Sin embargo, disponer de la información de las condiciones de salud y demográfica utilizada en su construcción es el pilar fundamental para poder realizar dicho análisis de riesgo.

Esta matriz fue trabajada para preparar la documentación para el Comité Internacional de Expertos (CIE) de la OPS con el propósito de certificar la interrupción de la transmisión endémica de sarampión, rubéola y síndrome de la rubéola congénita (SRC) en Chile (11). Inicialmente, se basó en el método propuesto por la OPS en la reunión auspiciada por ella en Nicaragua, en mayo de 2013 (Implementación y validación de los protocolos de evaluación del sistema de vigilancia del S/R/SRC y MRV), para identificar zonas de potenciales brotes de sarampión o rubéola asociados con la importación de casos frente a la eventualidad de que uno de estos virus fuera introducido por viajeros. Por ello, en una primera fase, las 15 Secretarías Regionales Ministeriales de Salud (SEREMI) del MINSAL la completaron a partir de respuestas dicotómicas (sí o no). Posteriormente, los resultados se consolidaron en el MINSAL para realizar una primera jerarquización de comunas y regiones con zonas de mayor o menor riesgo y construir mapas, que se publicaron en un documento técnico en 2014 (11).

### Propuesta de la nueva versión de la matriz de riesgo

Para esta nueva versión, el equipo técnico del Departamento de Epidemiología del MINSAL trabajó en una asesoría externa realizada por geógrafos con una duración estimada de aplicación de 14 meses (de octubre de 2014 a diciembre de 2015). Para agrupar las variables se dispuso de información procedente de fuentes directas del Programa Nacional de Inmunizaciones (PNI) y del Departamento de Estadísticas e Información en Salud (DEIS) del MINSAL, así como de otras fuentes oficiales y públicas que tienen información espacial (Ministerio de Obras Públicas, Servicio Nacional de Turismo, Instituto Nacional de Deportes, Ministerio de Bienes Nacionales).

En la fase de prueba, se realizó un estudio piloto en tres de las quince regiones de Chile correspondientes a zonas geográficas distintas entre sí (región de Tarapacá (norte), región Metropolitana de Santiago (centro) y región de Los Lagos (sur)) concentrando como unidades de análisis 89 comunas de las 346 que abarca todo el país. Con los primeros resultados se elaboró la “Guía metodológica” en las siguientes etapas: (A) selección de variables y construcción de bases de datos, (B) estandarización de variables, (C) ponderación de variables (con criterios de expertos), (D) cálculo del índice y espacialización del riesgo, y (E) transferencia y operatividad de la herramienta a equipos técnicos. Los procesos de estas etapas se detallan a continuación.

### Selección de variables y construcción de la base de datos

Con la matriz de riesgo propuesta por la OPS, las variables se seleccionaron y adecuaron a partir de diferentes fuentes de información secundaria pública disponibles en las comunas. Estas variables se agruparon en tres dimensiones: biológica, programática y demográfica.

La dimensión biológica está compuesta por la cobertura de vacuna triple viral (que contiene los antígenos del sarampión, papera y rubéola (SPR) de niños y niñas de 1 y 6 años de edad), y los resultados obtenidos a escala regional con el estudio de seroprevalencia de sarampión y rubéola utilizando muestras de la seroteca derivada de la Encuesta Nacional de Salud de Chile, 2009-2010. La dimensión programática contiene indicadores que evalúan el funcionamiento del programa de inmunizaciones y de vigilancia epidemiológica, como la tasa de deserción de vacunación y el silencio epidemiológico, respectivamente. La dimensión demográfica apunta a las condiciones socio-territoriales de las unidades comunales, que se agruparon en las siguientes variables: población, aislamiento, flujo fronterizo, recreación, comercio, zona de interés turístico, y ecoturismo. Cada dimensión está compuesta por las variables que aparecen en el cuadro 1.

**CUADRO 1.** Detalle de las variables y fuentes de información en la dimensión biológica, programática y demográfica

Dimensión	Variable	Fuente
Biológica	Cobertura de vacunación triple viral de 1 año (%)	Programa de Inmunización, MINSAL (registros validados en 2013)
	Cobertura de vacunación triple viral de 6 años (%)	
	Seroprevalencia de sarampión y rubéola (%)	Encuesta Nacional de Salud, 2010
Programática	Tasa de deserción (%)	Programa de Inmunización, MINSAL (registro de 2013 y 2014)
	Silencio epidemiológico (No.)	Departamento de Epidemiología, MINSAL (registro de 2010 a 2013)
Flujo migratorio	Pasos fronterizos (terrestre y aéreo)	Unidad de pasos fronterizos del Ministerio del Interior y Seguridad Pública (registro de 2013)
Aislamiento	Índice de aislamiento	Subsecretaría de Desarrollo Regional y Pontificia Universidad Católica de Chile (2009)
Población	Densidad de población	Instituto Nacional de Estadísticas (proyección de población para 2014)
	Población en zona urbana	Encuesta de Caracterización Socioeconómica Nacional, Ministerio de Desarrollo Social (2009)
Recreación	Canchas	Instituto Nacional del Deporte (registro de 2014)
	Museos	Consejo Nacional de Cultura y Artes (registro de 2014)
	Piscinas	Instituto Nacional del Deporte (registro de 2014)
	Centros culturales	Consejo Nacional de Cultura y Artes (registro de 2014)
	Bordes costeros	Ministerio de Obras Públicas (registro de 2014)
	Plazas y parques	Instituto Nacional de Estadísticas (registro 2014)
Demográfica	Caletas	Ministerio de Obras Públicas (registro 2014)
Comercio	Ferias y Persas	Confederación Gremial Nacional de Organizaciones de Ferias Libres (registro de 2014)
	Grandes almacenes	Cámara Chilena de Centros Comerciales (registro de 2014)
	Mercados	Información secundaria (registro de 2014)
Zonas de interés turístico Ecoturismo	Humedales	Convención sobre los Humedales de Importancia Internacional (RAMSAR) (registro de 2014)
	Parques nacionales y reservas naturales	Ministerio del Medio Ambiente (registro de 2014)
	Zona típica	Consejo de Monumentos Nacionales de Chile (registro de 2014)
	Zonas de interés turístico	Servicio Nacional del Turismo (registro de 2013)
	Rutas patrimoniales	Ministerio de Bienes Nacionales (registro de 2014)
	Puertos	Dirección Meteorológica de la Armada (registro de 2014)

***Fuente:*** Elaboración propia a partir de datos del Departamento de Epidemiología, División de Planificación Sanitaria (DIPLAS), Ministerio de Salud de Chile.

En este proceso, para la dimensión biológica y la programática se utilizaron bases de datos del MINSAL. Para la dimensión demográfica se hicieron las definiciones operativas: (A) definición formal de comuna urbana y rural, que está estandarizada por el INE y en la cual cada comuna, considerada como unidad de análisis, comprende localidades urbanas y entidades rurales; (B) definición de zonas de frontera con alto tránsito en nuestro país considerando los dos principales medios de transporte para dar cuenta de un flujo de personas que transiten por ellas; (C) establecimiento de la forma de medición de áreas periféricas densamente pobladas mediante la densidad de población (este ítem se relacionó con la concentración de bienes y servicios, aunque esto último está contenido en la construcción del índice de aislamiento comunal, otra variable considerada en esta dimensión de la matriz); (D) para las zonas industriales o de comercio y de recreación o eventos masivos (como figuran en la matriz original de la OPS), se contabilizaron los metros cuadrados de superficie comunal con el instrumento de planificación que establece las normas de uso de los suelos urbanos, aunque la escasez de información oficial derivó en la geolocalización de los principales hitos de comercio y de recreación por comuna.

**FIGURA 1.**
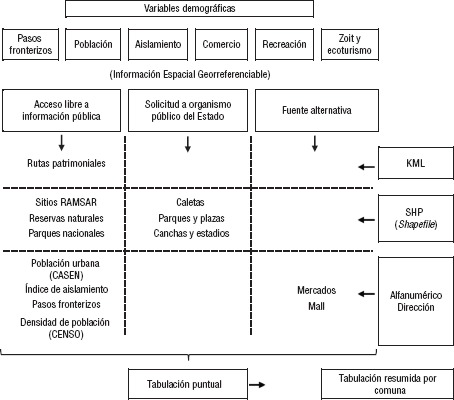
Elaboración de variables pertenecientes a la dimensión demográfica

Respecto a la unidad de análisis, el territorio se debe entender como una combinación de un componente geométrico y otro alfanumérico (12), unidos por un identificador único e irrepetible (Código Único Territorial). El primero de ellos hace referencia a un formato vectorial (punto, línea o polígono) con el cual se describen los objetos según su localización espacial, forma geométrica y tamaño, y el segundo proporciona información de las características temáticas o descriptivas. Los sistemas de información geográfica (SIG o GIS en inglés) permiten unir componentes geométricos y alfanuméricos con herramientas de unión espacial y ofrecen información territorial tabulada. A modo de ejemplo, para trabajar con las variables de la dimensión demográfica fue necesario construirlas en tres etapas (figura 1), que se describen a continuación.

***Fuente:*** Elaboración propia a partir de datos del Departamento de Epidemiología, División de Planificación Sanitaria (DIPLAS), Ministerio de Salud de Chile.

Etapa 1. Búsqueda de información en diferentes fuentes: información pública y oficial de bases de datos espaciales y otras alternativas. Para consolidarlos, dicha información se procesó y homogeneizó mediante un SIG con el fin de obtener datos espaciales a escala comunal.

Etapa 2. Generación de coordenadas geográficas a partir de archivos en formato KMZ – KML para la transformación a *shapefile* utilizando el sistema de coordenadas UTM, Datum WGSS84, Huso 19 Sur. Posteriormente, se sintetizó la información en una planilla a escala comunal. Por ejemplo, para la dimensión demográfica, con variables de tipo lineal y poligonal (rutas patrimoniales, parques nacionales y reservas naturales), el tratamiento de datos es diferente, ya que la extensión o la superficie de un único elemento traspasa los límites comunales y exige identificar cada comuna. Por otra parte, las variables que tienen la dirección física (nombre de la calle y numeración) fueron georreferenciadas manualmente con Google Maps.

Etapa 3. Generación de planillas tabuladas para tener la información sistematizada por cada variable junto con las coordenadas de la información complementaria, como el nombre de la comuna, el Código Único Territorial, la región, el ítem, el nombre de la variable o la fuente de información. Con cada tabla se diseñó la base de datos de las variables.

### Estandarización de variables

La estandarización tiene como objetivo permitir la comparación de variables expresadas en magnitudes diferentes, como ocurre, por ejemplo, al comparar una variable de dimensión biológica, como la cobertura de vacunación (porcentaje), con otra de dimensión demográfica, como las áreas de recreación (cantidad de áreas). Cada una de las variables se asocia con su dato numérico y a continuación cada valor se estandariza mediante puntaje omega con la siguiente fórmula (13):

$$Ev_{i}=\frac{X_{1}-m}{M-m}$$

donde:

EVi = estandarización de la variable en la i-ésima comuna

Xi = valor de la variable analizada para la unidad espacial

M = valor más desfavorable (negativo) de la variable

m = valor más favorable (positivo) de la variable

El resultado de la estandarización permite que los datos fluctúen en un rango de 0 a 1 según el cual cuanto más cercano a 0 son los resultados, más favorables son y, por el contrario, cuanto más cercanos a 1, más desfavorables. En primera instancia, las variables se analizaron para asignarles sus valores máximo y mínimo de tal forma que dicha asignación se realice con un criterio cuyo resultado pueda ser clasificado como favorable (menor riesgo) o desfavorable (mayor riesgo) en relación con la generación de brotes. Como ejemplo de la dimensión programática puede citarse la variable silencio epidemiológico: si se tienen 1 o más casos notificados de sarampión o rubéola en un período de tres años, el riesgo es menor, por lo que el resultado de la estandarización es cercano a 0 (favorable), y si no hay casos notificados en el mismo período, el riesgo es mayor y el resultado de la estandarización es 1 (desfavorable).

### Ponderación de variables

La asignación de valores asociados con el grado de importancia de cada variable del estudio se realizó aplicando el método del Proceso Analítico Jerárquico (Analytic Hierarchy Process o AHP), una herramienta matemática empleada para resolver problemas complejos con múltiples criterios (14). Esta herramienta funciona realizando comparaciones de variables una a una (biunívocas), utilizando escalas verbales referidas a criterios de valoración, con mínimos y máximos, y permite a los expertos fijar con facilidad niveles de clasificación respecto a la importancia de las variables sin condicionar su agrupación primaria (dimensión). Estas comparaciones se aplican a una matriz que remplaza las expresiones verbales por valores numéricos y establece un rango entre cada criterio o variable en el total de las dimensiones evaluadas.

Para ello, se convocó a un panel de expertos con 18 profesionales, 4 internacionales (red OPS) y 14 nacionales. Estos últimos representaban el ámbito nacional y regional de la red MINSAL, sociedades científicas y universidades. Todos ellos tenían un nivel elevado de conocimiento y de dominio del problema y experiencia en las áreas clínica, de vigilancia, inmunizaciones y del laboratorio en sarampión y rubéola. Esta técnica requiere un facilitador que pueda traducir este conocimiento de forma explícita en probabilidades y obtener buenas estimaciones en ausencia de cifras exactas, que deben ser ratificadas con el paso del tiempo a medida que se dispone de más información científica (15).

Para asignar valores asociados con el grado de importancia de cada variable (respuesta al *¿cuánto más importante?*), se utilizó una escala numérica relacionada con una escala verbal, que tiene diferentes interpretaciones según la importancia de las variables analizadas. Por ejemplo, con respecto al riesgo de importación del virus sarampión o de la rubéola, ¿cuál variable es más importante, cobertura de vacunación triple viral o áreas de recreación? Si la respuesta ha sido cobertura de vacunación triple viral (SPR), ¿cuánto más importante es la cobertura de vacunación sobre las áreas de recreación?

**FIGURA 2.**
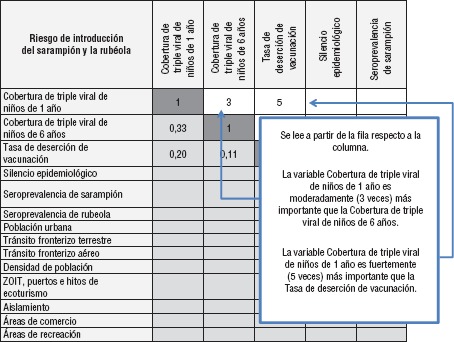
Ejemplo de lectura de la comparación biunívoca

La escala verbal especificada se transfiere a una matriz (figura 2) donde se aplica desde cada fila en relación con una columna. Como la variable B se compara con ella misma, siempre se le asignará el valor 1. Luego, respecto a las variables B y C, se aplican las preguntas “*Con respecto al objetivo planteado (A), ¿Cual es más importante, B o C?” “Si la respuesta ha sido B: ¿Cuánto más importante es B que C?”* y se asigna el valor entero de la escala verbal a la fila de la variable B (valor 3) y su fracción en relación con un entero en la celda inversa, es decir, en la fila de la variable C en relación con la variable B (valor 1/3).

***Fuente:*** Elaboración propia a partir de datos del Departamento de Epidemiología, División de Planificación Sanitaria (DIPLAS), Ministerio de Salud de Chile.

De esta manera, se va completando la matriz de comparación biunívoca y las variables más importantes se relevan al final del proceso según su ponderación final (cuadro 2). (El detalle del proceso del panel de expertos se encuentra detallado en la Guía Metodológica, disponible en http://epi.minsal.cl) La escala numérica puede ser igual a 1 (ambos elementos tienen la misma importancia), a 3 (importancia moderada de un elemento sobre otro), a 5 (fuerte importancia de un elemento sobre otro), a 7 (importancia muy fuerte de un elemento sobre otro), o a 9 (importancia extrema de un elemento sobre otro). Los valores 2-4, 6 y 8 se utilizan para medir juicios intermedios.

### Cálculo del índice y elaboración de mapas de riesgo

Con los datos ya estandarizados, se procede a multiplicar estos valores por la ponderación de cada variable según la importancia relativa establecida por el grupo de expertos (figura 2) y se obtiene el valor final que da cuenta del índice de riesgo, que se calcula con la siguiente ecuación:

$$VUT_{i}=\sum_{i=1}^{n}\left(v_{i}*p_{i}\right)$$

donde:

*VUT*_*i*_ = valor unidad territorial para la j-ésima comuna

*v*_*i*_ = i-ésima variable estandarizada

*p*_i_ = i-ésima ponderación asociada a la i-é-sima variable

j = 1, …, n-comunas i= 1,…, n-variables

Con los resultados obtenidos se representa en el espacio el índice de riesgo mediante la generación de mapas y, de esa forma, se identifican las comunas de mayor riesgo. Los resultados se clasifican según los valores del índice de riesgo en tres intervalos iguales, semejantes a los colores del semáforo, donde el verde representa la condición más favorable (riesgo bajo), el amarillo, la transición (riesgo medio) y el rojo, el más desfavorable (riesgo alto).

La confección de mapas se genera a partir del sumatorio de las ponderaciones de expertos, y para su elaboración se utiliza un SIG, como, por ejemplo, QuantumGIS, un programa informático de código abierto (está disponible en http://www.qgis.org/es/site/).

### Transferencia y operatividad de la herramienta a equipos técnicos

Cuando se completó la matriz a nivel regional con su correspondiente base de datos en 2015, se capacitó a los encargados regionales de las SEREMI de salud transfiriéndoles la información de todo el proceso. Esto incluyó la elaboración de las variables, la incorporación y edición de la base de datos regionales, la estandarización, la ponderación asignada por los expertos, y el cálculo del índice de riesgo y su interpretación numérica y gráfica con la elaboración de un mapa.

## RESULTADOS

**FIGURA 3.**
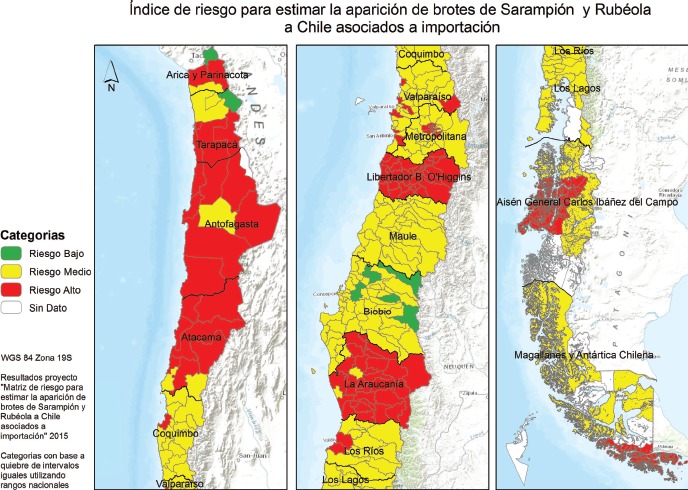
Resultado de la aplicación de la matriz de riesgo de brotes de sarampión y rubéola asociados con la importación de casos a Chile, 2013

La información obtenida en la matriz de riesgo se puede analizar desde distintos ámbitos. El primero es el grado de cumplimiento cuyo objetivo es identificar zonas de potenciales brotes de sarampión o rubéola asociados con la importación de casos. Los resultados obtenidos en este ámbito fueron los siguientes. De las 346 comunas de Chile analizadas, 34% se encontraba en el intervalo de riesgo alto, 59%, en el intervalo de riesgo medio, y 3%, en el intervalo de riesgo bajo. El porcentaje restante corresponde a comunas de las cuales no se dispuso de datos en al menos una de las trece variables requeridas para el cálculo del índice de riesgo (figura 3).

En las regiones de la zona norte se observó una tendencia de riesgo alto, en la zona sur, de riesgo medio, y Biobío fue la única región con riesgo bajo en mayor medida que el resto. El tercio del total de comunas del país en condición de riesgo alto presentó un mismo patrón de bajas coberturas de vacunación y de altos índices en la tasa de deserción, variables que presentan alta ponderación con relación al resto de variables.

**CUADRO 2.** Ponderación de las variables según la dimensión por el panel de expertos

Dimensión	Variables	Ponderación final	Porcentaje
Biológica	Cobertura de triple viral de 1 año	0,20	20
	Cobertura de triple viral de 6 años	0,16	16
	Seroprevalencia	0,11	11
Programática	Tasa de deserción de vacunación	0,13	13
	Silencio epidemiológico	0,06	6
Demográfica	Población urbana	0,04	4
	Tránsito fronterizo terrestre	0,03	3
	Tránsito fronterizo aéreo	0,05	5
	Densidad de población	0,03	3
	Zonas de interés turístico, puertos e hitos de ecoturismo	0,07	7
	Aislamiento	0,02	2
	Áreas de comercio	0,04	4
	Áreas de recreación	0,05	5
	Total	1,00	100

***Fuente:*** Elaboración propia a partir de datos del Departamento de Epidemiología, División de Planificación Sanitaria (DIPLAS), Ministerio de Salud de Chile.

**Fuente:** Departamento de Epidemiología, División de Planificación Sanitaria (DIPLAS), y Ministerio de Salud de Chile.

Por otro lado, se identificó un número muy bajo de comunas en condición de riesgo bajo (3%) con un mismo patrón de altas coberturas en al menos una de las vacunas (registros mayores a 100%), baja tasa de deserción (principalmente resultados negativos de la tasa), comunas con densidad de población muy baja, alta población en la zona rural, principalmente aislada, con bajo flujo de personas en tránsito terrestre, y bajo número de áreas comerciales y de recreación.

El segundo ámbito de análisis es la utilización como herramienta metodológica del análisis epidemiológico y de apoyo a la toma de decisiones en la tarea de prevenir brotes de sarampión o rubéola asociados con la importación de casos. Los resultados fueron los siguientes: el desarrollo de la matriz de riesgo está basado en registros a nivel nacional para evaluar el territorio con los mismos parámetros en el momento en que se toman decisiones de prevención y control. No obstante, con la transferencia metodológica a las distintas SEREMI de salud se identificó la realidad local y se apreció una dinámica diferente de la de los resultados nacionales. Cada una de las regiones cuenta con categorizaciones diferenciadas que no son comparables entre regiones, aunque sí analizables internamente para fines de implementación metodológica y la elaboración de estrategias regionales. Esta herramienta se basa en un método robusto de construcción de información socioterritorial, validada y ponderada por expertos en sarampión, rubéola y salud pública en diferentes niveles de toma de decisiones.

## DISCUSIÓN

Este proyecto conjunto entre el Ministerio de Salud de Chile y la OPS para lograr avances metodológicos se realizó gracias al trabajo de epidemiólogos, geógrafos, estadísticos, demógrafos y otros profesionales con experiencia en estas enfermedades, que unificaron criterios trabajando como equipo multidisciplinario.

Las variables de la matriz de riesgo fueron reevaluadas con criterios técnicos por el equipo chileno y el panel de expertos en la materia, a partir del modelo presentado por la OPS en 2013. La mayoría de las variables se mantuvo en los análisis, fueron adecuadas según la disponibilidad de información a niveles comunales, se agruparon en las distintas dimensiones mostradas y se ponderaron según su nivel de importancia con los métodos propuestos.

La dimensión biológica es la más relevante y al ponderarse representa la mitad del total de importancia, ya que apunta hacia el grado de protección de la población (inmunidad de rebaño o *heard immunity*) frente a un riesgo potencial de importación de sarampión o rubéola. La dimensión programática refleja el cumplimiento de programas de inmunizaciones y la necesidad de disponer de una vigilancia activa que permita capturar oportunamente los casos importados o los brotes asociados con ellos. Las variables de la dimensión demográfica son un indicador de los determinantes de la población que pueden llegar a influir en los estados de salud individual y colectiva, aunque sea difícil intervenir en ellos tanto desde el sector salud como desde otros ámbitos.

En general, los resultados de las variables estandarizadas y ponderadas indican que en las respectivas comunas de las regiones de Chile el riesgo de tener brotes postimportación es medio o alto. Esto requiere mejorar los indicadores de las variables utilizadas, como en las dimensiones biológicas y programáticas, que, según el método empleado en este estudio, se aproximan a valores de mayor riesgo y pueden mejorarse, por ejemplo, en áreas tales como las coberturas bajas de vacunación o los lugares con silencio epidemiológico.

Durante la última fase del proyecto, el método se transfirió a las regiones, junto con todos los procesos de su desarrollo hasta el análisis de sus resultados. La mayoría de los participantes señaló incongruencias con algunos datos de la dimensión demográfica, ya que las bases de datos utilizadas según fuentes definidas no reflejaban la realidad regional. No obstante, si estas bases se actualizan con datos disponibles, representan solo un tercio de la ponderación total (33%).

La flexibilidad de la matriz permite introducir mejoras a nivel regional o nacional frente a cambios de los escenarios epidemiológicos y que los equipos locales la adapten a otros problemas de salud. Las bases de datos pueden actualizarse para calcular los índices de riesgo con mayor exactitud y todo ello es factible cuando se dispone de información fiable y oficial y se respetan los estándares metodológicos.

En este contexto, el método presentado contribuye a considerar los resultados globales y otros más específicos, lo que sumado al análisis espacial permitirá facilitar la comprensión del riesgo de importación a escala comunal y, con ello, identificar las zonas que se deben priorizar para ser intervenidas y mejorar los resultados sanitarios.

Las limitaciones para trabajar la matriz de riesgo son contar con recursos humanos cualificados y técnicos en la materia, con condiciones mínimas de manejo de bases de datos y de herramientas de análisis espacial, poder adaptarlas según las necesidades, y utilizar las fuentes de información actualizada en las diferentes categorías.

La matriz cumple con el objetivo de priorizar zonas de intervención a nivel local, pero es limitada para comparar riesgos entre territorios. Por consiguiente, sólo es útil cuando el equipo técnico multidisciplinario prioriza mejorar los resultados comunales reflejados en el mapa según los colores del semáforo mediante la elaboración de un plan que facilite la mejora de los índices alcanzados.

Se recomienda utilizar esta herramienta para el análisis de la información y que su uso se traduzca en un indicador de gestión de áreas donde puedan implementarse mejoras. Asimismo, se ha de elaborar un plan de intervención en las comunas de mayor riesgo, por ejemplo, desarrollando estrategias dirigidas a aumentar las coberturas de vacunación o a intensificar la vigilancia epidemiológica en zonas de alto riesgo. Por último, es preciso reforzar continuamente el plan de sostenibilidad de la eliminación de sarampión y rubéola en Chile, así como actualizar la matriz de riesgo, pues ello ayudará a afrontar mejor las amenazas permanentes de aparición de brotes de sarampión o rubéola asociados con la importación de casos mientras no se declare su erradicación mundial.

### Agradecimiento.

Los autores agradecen el apoyo permanente de los equipos técnicos de epidemiología e inmunizaciones de las Secretarías Regionales y del Ministerio de Salud, y del Subdepartamento de Enfermedades Virales del Instituto de Salud Pública, componentes esenciales para lograr la eliminación del sarampión y la rubéola en Chile.

### Financiación.

El contrato de los asesores externos que elaboraron la matriz de riesgo se realizó con fondos la OPS/OMS.

### Declaración.

Las opiniones expresadas por los autores son de su exclusiva responsabilidad y no reflejan necesariamente los criterios ni la política de la Organización Panamericana de la Salud o de la *RPSP/PAJPH* y/o de la OPS.
